# Maximum Power Point Tracking of Photovoltaic System Based on Reinforcement Learning

**DOI:** 10.3390/s19225054

**Published:** 2019-11-19

**Authors:** Kuan-Yu Chou, Shu-Ting Yang, Yon-Ping Chen

**Affiliations:** Institute of Electrical and Control Engineering, National Chiao Tung University, Hsinchu 30010, Taiwan; ystfc72.eed02@nctu.edu.tw (S.-T.Y.); ypchen@cc.nctu.edu.tw (Y.-P.C.)

**Keywords:** maximum power point tracking (MPPT), photovoltaic (PV) system, reinforcement learning, Q-learning, Q-network

## Abstract

The maximum power point tracking (MPPT) technique is often used in photovoltaic (PV) systems to extract the maximum power in various environmental conditions. The perturbation and observation (P&O) method is one of the most well-known MPPT methods; however, it may face problems of large oscillations around maximum power point (MPP) or low-tracking efficiency. In this paper, two reinforcement learning-based maximum power point tracking (RL MPPT) methods are proposed by the use of the Q-learning algorithm. One constructs the Q-table and the other adopts the Q-network. These two proposed methods do not require the information of an actual PV module in advance and can track the MPP through offline training in two phases, the learning phase and the tracking phase. From the experimental results, both the reinforcement learning-based Q-table maximum power point tracking (RL-QT MPPT) and the reinforcement learning-based Q-network maximum power point tracking (RL-QN MPPT) methods have smaller ripples and faster tracking speeds when compared with the P&O method. In addition, for these two proposed methods, the RL-QT MPPT method performs with smaller oscillation and the RL-QN MPPT method achieves higher average power.

## 1. Introduction

Sustainable energy such as solar energy is often seen as one of the solutions to reduce pollution caused by thermal power generation. A photovoltaic (PV) module is able to convert solar energy into electrical energy without generating greenhouse gases and coal dust, and it is wildly used since the deployment is relatively easy compared to other sustainable energy sources such as tidal energy and biogas energy. However, low efficiency is the main drawback of a PV system. Therefore, several maximum power point tracking (MPPT) methods are proposed in order to extract maximum power from the PV module. The perturbation and observation (P&O) method is one of the most common MPPT methods and can be implemented model-free [1,2]. However, a large size perturbation setting will lead to large oscillations near the maximum power point (MPP), while a small step size perturbation setting will slow down the tracking speed. Therefore, several adaptive methods are proposed to improve the P&O method [3,4,5]. The adaptive P&O method basically modifies the step size based on the amount of the power difference between two perturbations. However, to achieve the best performance, the ratio between the step size and power difference needs to be tuned according to the actual model. Additionally, several fuzzy P&O methods are proposed to perform the MPPT [6,7]. Although fuzzy logic control is a model-free control method, expert knowledge is required when designing the fuzzy parameters.

Capable of performing model-free control, reinforcement learning (RL) [8] is widely used in solving control problems because it can learnt by interacting with the system without prior knowledge of the system model. There are two similar MPPT methods based on RL for PV system proposed in [9] and [10], and a Markov decision process (MDP) is used as the framework to describe the problem. The states are defined by the moving direction and the position of the operating point relative to the MPP. The action is the choice of variable step sizes, and the reward is defined as the power difference before and after the action is taken. Kofinas et al. [11] also proposed a solar MPPT method based on RL. The discretized current and voltage value and a parameter of judging whether the operating point is at MPP are used as the system description. The perturbation step size can be chosen appropriately according to the interacting experience with the system. The MPPT method in [11] is similar to one of the proposed methods, reinforcement learning-based Q-table maximum power point tracking (RL-QT MPPT), however, it is designed to be learnt online, which causes several oscillations during the tracking process. In addition, the above mentioned solar MPPT methods approached by RL are all implemented by constructing the Q-table, which may lead to the problem of generalization representation. Using a neural network as an approximation of the Q-table was first introduced by [10], and the experience replay technique is also proposed by [12]. Mnih et al. [13] proposed a fixed target Q-network technique to stabilize the training process. In this paper, two RL MPPT methods are proposed by using the Q-learning algorithm. One constructs the Q-table (RL-QT MPPT) and the other adopts the Q-network (RL-QN MPPT). These two proposed methods do not require the information of an actual PV module in advance and can track the MPP through offline training in two phases, the learning phase and the tracking phase. From the simulation and experimental results, it is expected to outperform the P&O method since the step size can be chosen according to the learned perturbation experience.

At first, this paper will introduce the model of the PV module in Section 2. Then the P&O method and proposed reinforcement learning-based MPPT methods are described in Section 3. In Section 4, the simulation and experimental results show the comparison of the traditional P&O method and the proposed methods to prove its performance. Finally, the conclusion is given in Section 5.

## 2. Model Description of PV Module

When a PV module is exposed to the sunlight, the electrons inside it will absorb the energy and jump to a higher energy state. Some free mobile electrons will be released through a connected wire and thus form a current. This phenomenon is known as the photovoltaic effect [14].

**Figure 1:**
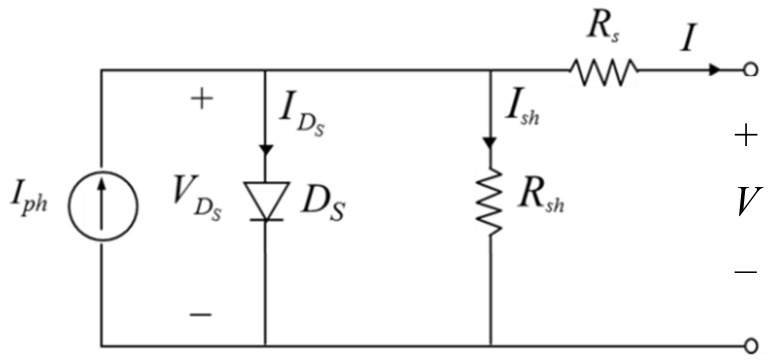
Single-diode model of a solar cell.

A solar cell is a device to generate electrical power based on the photovoltaic effect. Figure 1 shows the single-diode model [15] of a solar cell, where $I_{ph}$ is the photo-generated current, $I_{D_{S}}$ is the current through the diode $D_{s}$, $V_{D_{S}}$ is the voltage across $D_{s}$. $I_{sh}$ is the current through shunt resistor $R_{sh}$, and *I* is the current through series resistor $R_{s}$. In addition, *I* and *V* are the output current and output voltage of the solar cell, respectively.

According to Kirchhoff’s current law, the output current can be expressed as

(1) $$I=I_{ph}-I_{D_{S}}-I_{sh}=I_{ph}-I_{D_{S}}-\frac{V+IR_{S}}{R_{sh}}.$$

The current $I_{D_{S}}$ can be expressed by the Shockley equation below:

(2) $$I_{D_{S}}=I_{0}\left(e^{\frac{qV_{D_{S}}}{\eta KT}}-1\right)=I_{0}\left(e^{\frac{q\left(V+R_{S}I\right)}{\eta KT}}-1\right)$$

where $I_{0}$ represents the reverse saturation current, *q* is the elementary charge, *K* is Boltzmann’s constant, *T* is the temperature and η is the diode ideality factor. Note that η=1~2 and η=1 for an ideal diode [16].

Substituting (2) into (1) yields the *I-V* characteristic expression of a solar cell model shown as

(3) $$I=I_{ph}-I_{0}\left(e^{\frac{q\left(V+R_{S}I\right)}{\eta KT}}-1\right)-\frac{V+IR_{S}}{R_{sh}}.$$

The photo-generated current $I_{ph}$ is affected by solar irradiance *S* and environment temperature *T* as below:

(4) $$I_{ph}=\frac{S}{1000}\left[I_{scr}+K_{i}\left(T-T_{r}\right)\right]$$

where $I_{scr}$ is the short circuit current, $K_{i}$ is the temperature coefficient of the short circuit current, and $T_{r}$ is the reference temperature.

**Figure 2:**
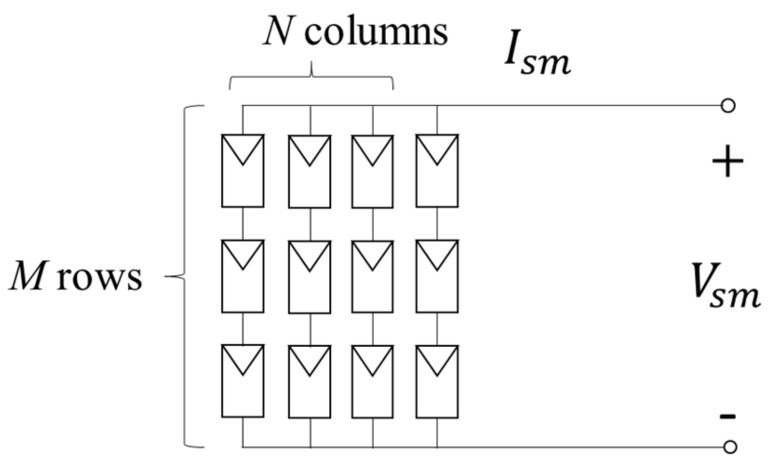
Equivalent circuit of a photovoltaic (PV) module.

A PV module usually consists of several solar cells in series or in parallel, i.e., composed of *M* rows and *N* columns of solar cells as depicted in Figure 2. The output voltage $V_{sm}$ of *M* solar cells in series and the output current $I_{sm}$ of *N* solar cells in parallel are respectively given as

(5) $$V_{sm}=MV$$

(6) $$I_{sm}=NI.$$

Substituting (5) and (6) into (3) yields:

(7) $$I_{sm}=NI_{ph}-NI_{0}\left(e^{\frac{q\left(\frac{V_{sm}}{M}+R_{s}\frac{I_{sm}}{N}\right)}{\eta KT}}-1\right)-\frac{\frac{N}{M}V_{sm}-I_{sm}R_{s}}{R_{sh}},$$

which is the *I–V* expression of an M×N PV module.

**Figure 3:**
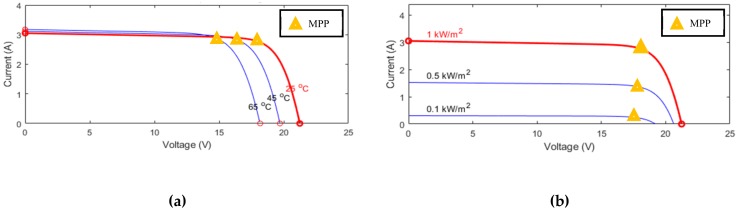
*I–V* curves of a PV module with (**a**) different temperature and (**b**) different irradiance.

As described in (7), the *I–V* curve of a PV module is strongly affected by environmental factors, including the variance of the solar irradiance and the module temperature. Figure 3a shows the *I–V* curves with different temperatures under the same irradiance condition. As the temperature rises, the curve moves left and vice versa. In addition, the plot of *I–V* curves under several distinct irradiance conditions with a constant temperature is presented in Figure 3b. It is obvious that the escalation of solar irradiance will cause a raise in the *I–V* curve, correspondingly. From Figure 3a,b, the maximum power point changes as the irradiance and module temperature vary, thus the variance of irradiance and module temperature will be considered in the proposed MPPT.

## 3. Maximum Power Point Tracking (MPPT) Control

**Figure 4:**
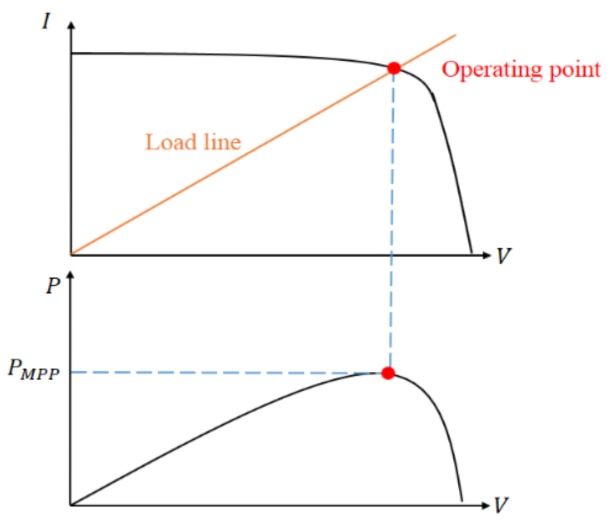
Load line and operating point.

As previously mentioned, the input impedance of the converter can be tuned by varying the duty ratio. If a PV module’s output impedance is matched with the converter’s input impedance, the maximum power can be extracted. As illustrated in Figure 4, the operating point of a PV module is the intersection of the *I–V* curve and the load line. The purpose of maximum power point tracking (MPPT) is to reach the operating point where the PV module has maximum power output. In this paper, the MPPT is implemented by adjusting the duty ratio of the converter. The P&O method will be discussed in Section 3.1, and the RL MPPT methods will be proposed in Section 3.2.

### 3.1. Perturbation and Observation (P&O) Method

**Figure 5:**
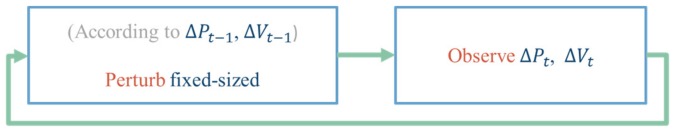
Concept of the perturbation and observation (P&O) method.

Figure 5 briefly illustrates the concept of the perturbation and observation method (P&O method), which is one of the well-known tracking methods. At time *t*, a fixed-sized perturbation is performed according to the measured power difference $∆P_{t-1}$ and voltage difference $∆V_{t-1}$. Then the change of the power and the voltage will be observed. With a new power difference $∆P_{t}$ and a new voltage difference $∆V_{t}$, the system can be perturbed accordingly.

**Figure 6:**
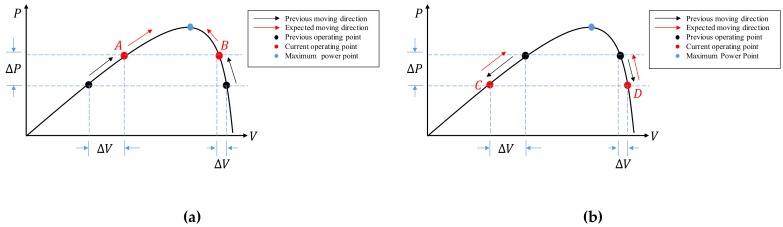
(**a**) Positive power difference condition; (**b**) negative power difference condition.

To drive the operating point toward the MPP, the system first measures the present power $P\left(n\right)$ and voltage $V\left(n\right)$ and then calculates the difference of power and the difference of voltage. If ∆P>0, there are two cases, operating points A and B, as depicted in Figure 6a. To move toward the MPP, the duty ratio should be decreased for point A with ∆V>0, and increased for point B with ∆V<0.

On the other hand, if ∆P<0, there are two cases, operating points *C* and *D*, as depicted in Figure 6b. To move toward the MPP, the duty ratio should be decreased for point *C* with ∆V<0, and increased for point *D* with ∆V>0.

### 3.2. Design of the Reinforcement Learning MPPT System

Sequential decision-making is a common problem in real life, for example, an infant trying to walk by stretching a leg forward and move his body. By taking a series of actions, the infant will have a chance to reach his goal (keep moving forward). The Markov decision process (MDP) provides a systematic framework for describing this sequential decision-making problem. To solve an MDP, a reinforcement learning (RL) method is proposed by [8]. Learning by interacting with the environment is a human’s intuitive learning skill. When facing an unknown system, the human will interact with it according to their own understanding of the system, and then receive feedback. This feedback signal provides a criterion for judging how “good” or “bad” an action is under a specific circumstance. The actions with better outcomes will have a larger chance to be chosen, i.e., they are reinforced, while the actions with worse outcomes will have smaller chance of being done in the future.

**Figure 7:**
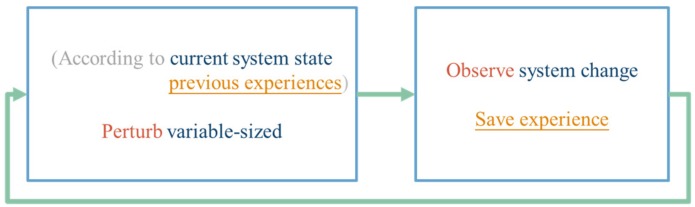
The concept of reinforcement learning-based maximum power point tracking (RL MPPT).

With the description of RL provided above, the concept of the variable step size tracking method based on RL is shown in Figure 7. The perturbation size can be chosen according to the observed system state and the previous experiences. Once the perturbation is done, the system change including the change of the state and the feedback signal will be observed and the interaction experience will be learned.

To provide a detailed description of the RL MPPT, the background knowledge of RL will be described from Section 3.2.1 to Section 3.2.5, including the description of the system, the interaction, and the evaluation of the interaction experience. Finally, the design of the RL MPPT methods, including the RL-QT MPPT method and the RL-QN MPPT method will be proposed in Section 3.3.

#### 3.2.1. Markov Decision Process and Reinforcement Learning Problem

A Markov decision process (MDP) [17,18] consists of *S*, *A*, *T*, and *R*, where *S* represents the set of environment’s state description, and *A* is the set of available actions the agent can take. *T* is the transition function, which indicates the system’s probability distribution of jumping from any state to all possible states after applying all available actions, and it is denoted as $T:S\times A\times S\to \left[0,1\right]$. For example, the probability of jumping from the state s∈S to state  s′∈S after applying the action α∈A can be written as $T\left(s,a,s\prime \right)$. For an MDP, the Markov properties hold. Consider a discrete-time series t=1,2,…, the next state $s_{t+1}$ only depends on the current state $s_{t}$ and the current action $a_{t}$, as shown below:

(8) $$P\left(s_{t+1}\left|s_{t},a_{t},s_{t-1},a_{t-1},\ldots \right.\right)=P\left(s_{t+1}\left|s_{t},a_{t}\right.\right)=T\left(s_{t},a_{t},s_{t+1}\right).$$

A reward *R* is a scalar numerical signal received from doing an action at a state, and the reward function *R* can be formally represented as R:S×A×S→ℝ.

**Figure 8:**
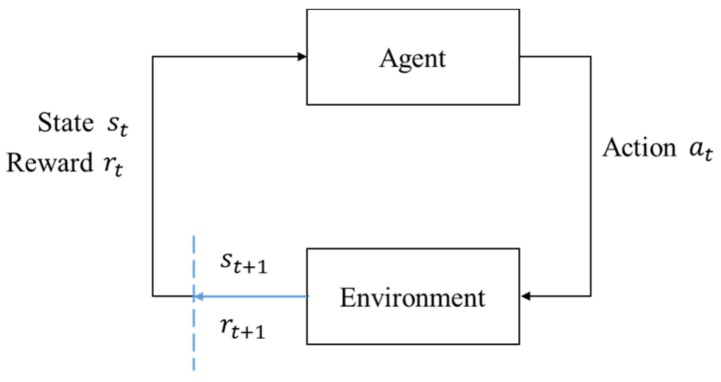
The agent–environment interface.

In RL, the one who actively takes actions that affect the environment is called an agent, and the environment is an object that reacts passively to the agent. The interaction between the agent and the environment can be described under the MDP framework. The agent–environment interface is shown in Figure 8. The agent and the environment interact discretely in a time series t=0,1,2,3. For each time step *t*, the description of the environment’s current condition $s_{t}$ is obtained by the agent. The agent will decide which action $a_{t}$ should be taken based on some rules. Then a numerical reward signal $r_{t+1}$ and a new state $s_{t+1}$ are brought out by the environment.

**Figure 9:**
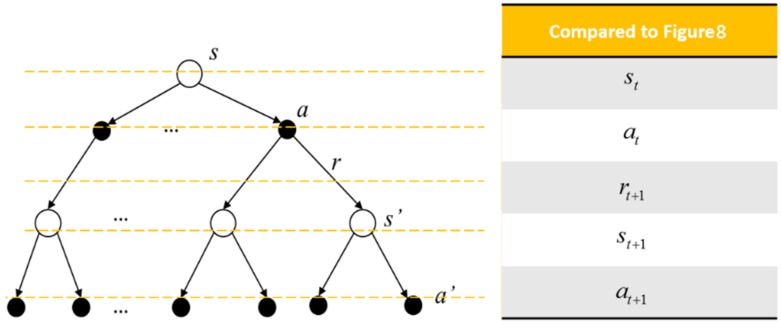
Sequential agent–environment interaction diagram.

Figure 9 is a common representation of describing the interaction between the agent and the environment. The white circles represent the states and the black dots are the actions. The top white circle is the current state, and the middle black dots are all of the available actions. The possible succeeding states are the white circles in the middle, and the corresponding available actions are listed at the bottom of the diagram.

**Figure 10:**
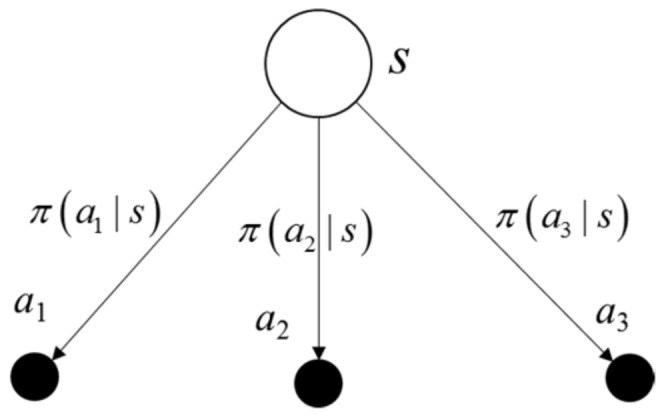
Example of the policy.

The rule followed by an agent is called a policy. Formally, an agent’s policy π is the mapping of the current state to the selection probability of the available actions. Hence, the probability of selecting $a_{t}$ when the agent is at the state $s_{t}$ can be denoted as $\pi_{t}\left(a_{t}|s_{t}\right)$. For example, under a policy  π, the probability of selecting $a_{1}$ at state *s* can be written as $\pi \left(a_{1}|s\right)$, as shown in Figure 10. With the experience (such as the tuple ($s_{t},a_{t},r_{t+1},s_{t+1}$)) gathered, the policy can be modified. The goal of reinforcement learning is to obtain a policy, such that the received cumulative rewards are maximized.

#### 3.2.2. Long Term Rewards

The expected return $G_{t}$ is the sum of the discounting rewards the agent expected to receive at time *t*, as below:

(9) $$G_{t}=r_{t+1}+\gamma r_{t+2}+\gamma^{2}r_{t+3}+\ldots =r_{t+1}+\gamma G_{t+1}$$

where γ is the discounting parameter, 0≤γ≤1. The rewards are discounted in order to avoid infinite cumulated rewards. With a smaller γ, the agent focuses on the recent rewards more, while a larger γ will make the agent be farsighted, and thus long term rewards will be considered. Also, the expected return can be written as an iterative form, which indicates that the current return is equal to the sum of the immediate reward $r_{t+1}$ and the successor state return $G_{t+1}$.

#### 3.2.3. Action Value and Optimal Action Value

The action value is the agent’s expected return starting from the state *s*, with the action a chosen and thereafter following the policy π, as described in (10):

(10) $$q_{\pi }\left(s,a\right)=E_{\pi }\left[G_{t}\left|s_{t}=s,a_{t}=a\right.\right]=\sum_{s^{\prime },r}p\left(s^{\prime },r\left|s,a\right.\right)\left[r+\gamma \sum_{a^{\prime }}\pi \left(a^{\prime }\left|s^{\prime }\right.\right)q_{\pi }\left(s^{\prime },a^{\prime }\right)\right]$$

where *r* is the immediate reward received by doing action a, and $\gamma \sum_{a^{\prime }}\pi \left(a^{\prime }\left|s^{\prime }\right.\right)q_{\pi }\left(s^{\prime },a^{\prime }\right)$ is the discounted action value starting from all possible next states $s^{\prime }$.

**Figure 11:**
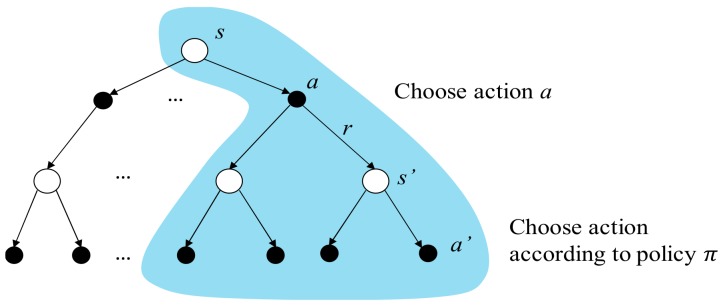
The backup diagram of action value function.

The backup diagram of the action value is shown in Figure 11. The top white circle and black dot are the state s and the action a being chosen under the state s, respectively. *r* is the reward received consequently. The white circles in the middle are the successor states $s^{\prime }$ the agent may obtain after doing action a. After that, the agent does actions according to the policy π, and the black dots in the bottom are the possible actions a′ being chosen.

**Figure 12:**
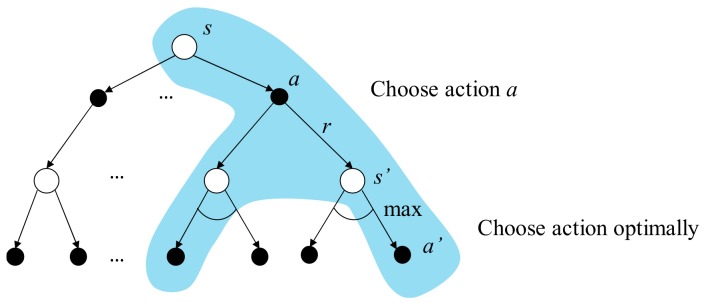
The backup diagram of the optimal action value function.

To an action value function, a policy π is better than the other policy $\pi^{\prime }$, or $\pi >\pi^{\prime }$, if and only if $q_{\pi }\left(s,a\right)\geq q_{\pi^{\prime }}\left(s,a\right),\forall s\in S,a\in A$. There always exists a policy that is better or equal to all the other policies and that is an optimal policy. The optimal action value function is defined as the expected return starting from the state s with the action a chosen, thereafter following the optimal policy, such that the expected returns in the following states are maximized, as shown in (11)

(11) $$q_{*}\left(s,a\right)=\underset{\pi }{\max}q_{\pi }\left(s,a\right)=\sum_{s^{\prime },r}p\left(s^{\prime },r\left|s,a\right.\right)\left[r+\gamma \;\underset{a^{\prime }}{\max}\;q_{*}\left(s^{\prime },a^{\prime }\right)\right]$$

where, $\underset{a^{\prime }}{\max}q_{*}\left(s^{\prime },a^{\prime }\right)$ is the optimal action value function of the next state $s^{\prime }$ with optimal action $a^{\prime }$ chosen. The backup diagram of the optimal action value function is depicted in Figure 12 with the blue background. The arc between different $a^{\prime }$ is a representation of choosing the optimal $a^{\prime }$ such that the return starting from $s^{\prime }$ is maximized.

#### 3.2.4. Introduction to Q-learning

**Figure 13:**
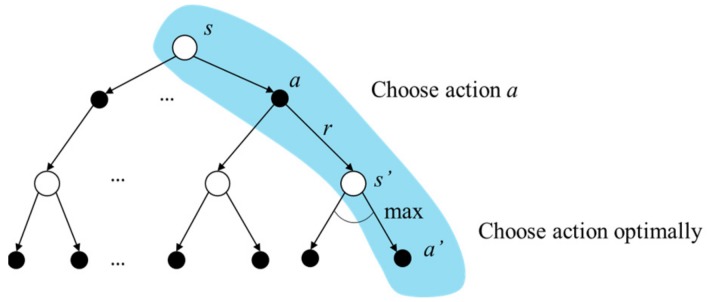
The backup diagram of Q-learning.

Q-learning [19] is a model free temporal difference (TD) method to perform RL. A Q-table is constructed through bootstrapping to store the optimal action value of any state-action pair. The one-step update of Q-learning is shown as below:

(12) $$Q\left(s,a\right)\leftarrow Q\left(s,a\right)+\alpha \left[r+\gamma \underset{a\_\in A\left(s^{\prime }\right)}{\max}Q\left(s^{\prime },a\_\right)-Q\left(s,a\right)\right]$$

where $Q\left(s,a\right)$ directly approximates the optimal action value q* and is independent of the policy followed. For the state s′, an optimal action a_ is expected to be selected within action set *A(s’)* so that the Q value at s′ can be maximized, i.e.,$\underset{a\_\in A\left(s^{\prime }\right)}{\max}Q\left(s^{\prime },a\_\right)$. The updating rate of the *Q* value is α, 0≤α<1, and *Q* has been proven to converge to q* with a probability 1 by [19]. The backup diagram of Q-learning is depicted in Figure 13 with a blue background. Through actual interactions, the experiences $\left(s,a,r,s\prime \right)$ can be acquired. According to the interaction experiences, the *Q* values can be updated by (12) and stored in a tabular form, which is called a Q-table.

Once the Q-table is fully constructed, the optimal policy can be extracted by greedily choosing the action with the largest optimal action value for each state, i.e., $\underset{a\in A\left(s\right)}{\arg\max}Q\left(s,a\right)$. Table 1 is an example of policy extraction from a well-constructed Q-table. The agent will take the action *A*3 when it is at the state *S*1 since $\underset{a\in \left\{A1,A2,A3\right\}}{\arg\max}Q\left(S1,a\right)=A3$. Similarly, *A*1 should be taken at states *S*2 and *S*4, and *A*2 should be taken at state *S*3.

One of the issues in RL is the exploration–exploitation trade-off [8]. For some RL problems, the policy changes with time, thus off-line learning is not suitable for them. Therefore, for each time step, the system is designed to be ε-greedy, i.e., choosing actions randomly to explore new possible rules in a probability ε and otherwise following the learned policy and always choosing the action with the largest Q value.

The algorithm of Q-learning is shown in Algorithm 1. First, the elements in the Q-table are initialized. The ε-greedy, the learning rate α and the discount factor γ are also initialized. Note that for a non-episodic MDP, there is no ending state, therefore, γ should be less than 1 to avoid infinite expected return.

The agent observes the current state first. With a probability ε, the agent will randomly choose the available actions, otherwise, it will choose the action with the largest Q value according to the Q-table. After applying the action, the environment will generate the reward signal $r_{t+1}$, and then a new state $s_{t+1}$ can be observed. With $\left(s_{t},a_{t},r_{t+1},s_{t+1}\right)$ obtained, the element $Q\left(s_{t},a_{t}\right)$ in the Q-table can be updated using (34). Finally, the current state $s_{t}$ is replaced by the new state $s_{t+1}$, and one step of an update is completed.

**Algorithm 1**: Non-episodic Q-learning algorithmInitialize $Q\left(s,a\right),\forall s\in S,a\in A$
Initialize ε,α,γ,0≤ε≤1,0≤α<1,0<γ<1
Observe $s_{t}$
**Repeat** (for each time step *t*)
$\;\;\left\{\begin{array}{cc} \mathrm{randomly}\;\mathrm{choose}\;a_{t} & \;\;\mathrm{probability}\;\varepsilon \\ a_{t}=\underset{a\_\in A\left(s_{t}\right)}{\arg\max}Q\left(s_{t},a\_\right) & \;\;\;\mathrm{otherwise} \end{array}\right.$
    Apply $a_{t}$, observe $r_{t+1}$ and $s_{t+1}$

$\;\;Q\left(s_{t},a_{t}\right)\leftarrow Q\left(s_{t},a_{t}\right)+\alpha \left[r_{t+1}+\gamma \underset{a\_\in A\left(s_{t+1}\right)}{\max}Q\left(s_{t+1},a\_\right)-Q\left(s_{t},a_{t}\right)\right]$

$\;\;s_{t}\leftarrow s_{t+1}$


**Figure 14:**
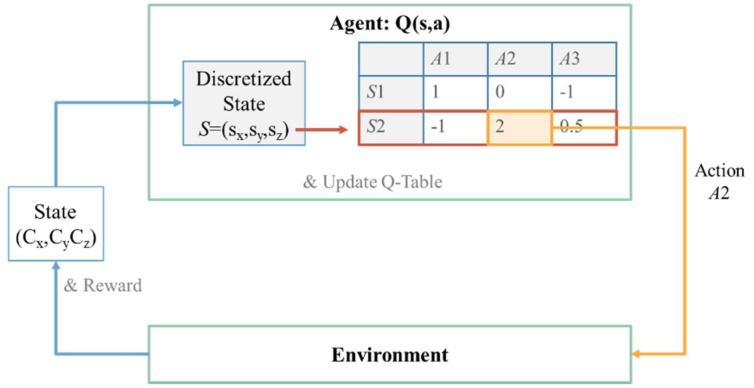
An example of agent–environment model with Q-table.

An example of the agent–environment model with the Q-table is illustrated in Figure 14. The state representation generated by the environment is required to be discretized to perform table lookup on the Q-table, which is impractical in some cases, such as the state space is too large or the state space is continuous. Therefore, an approximation of the Q-table using a neural network, named Q-network [13], has been implemented in this paper.

#### 3.2.5. Q-Learning with Neural Network Approximation

**Figure 15:**
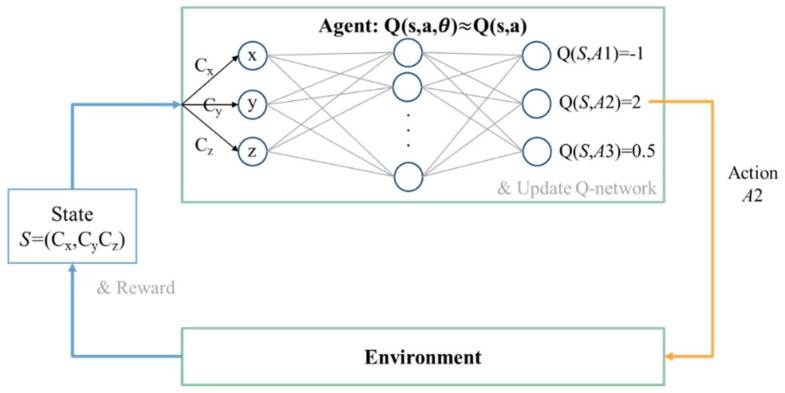
An example of the agent–environment model with the Q-network.

The Q-table in Figure 14 can be approximated as the Q-network in Figure 15. The state representation can be directly used as the input of the Q-network without discretization. The number of input nodes is the dimension of the state representation. The output of the Q-network $Q\left(s,a;\theta \right)$ is used to approximate the optimal action value q*, where θ is the weight of the network, i.e.,$Q\left(s,a;\theta \right)\approx q_{*}\left(s,a\right)$.

The loss function, also known as the cost function [20], of the Q-network in iteration i is defined as:

(13) $$L_{i}\left(\theta_{i}\right)={\left(\underset{\mathrm{target}\;Q\;\mathrm{value}}{\underset{︸}{y_{i}}}-\underset{\mathrm{estimate}\;Q\;\mathrm{value}}{\underset{︸}{Q\left(s,a;\theta_{i}\right)}}\right)}^{2}$$

where $y_{i}=r+\gamma \underset{a^{\prime }}{\max}Q\left(s^{\prime },a^{\prime };\theta_{i-1}\right)$ is the target Q value based on the current reward r and the maximum Q value at the next step S′ generated by the Q-network in iteration i−1, and $Q\left(s,a;\theta_{i}\right)$ is the estimated Q value provided by the Q-network in iteration i. For every i, (13) should be minimized in order to approximate the estimate Q value to the target Q value. The target Q-network parameter of the previous iteration, $\theta_{i-1}$, should hold fixed during the training in iteration i. This may cause the oscillation or divergence of the policy since the target Q value is affected immediately after updating the Q-network for every iteration.

To stabilize the learning process, a fixed target Q-network technique is proposed by [13], and the loss function is written as

(14) $$L_{i}\left(\theta_{i}\right)={\left(y_{i}-Q\left(s,a;\theta_{i}\right)\right)}^{2}={\left(\underset{\mathrm{target}\;Q\;\mathrm{value}}{\underset{︸}{\left(r+\gamma \underset{a^{\prime }}{\max}\hat{Q}\left(s^{\prime },a^{\prime };\theta^{-}\right)\right)}}-\underset{\mathrm{estimate}\;Q\;\mathrm{value}}{\underset{︸}{Q\left(s,a;\theta_{i}\right)}}\right)}^{2}$$

where $\theta^{-}$ is the old parameter several iterations before, and it will update to the current value for every $C_{T}$ iterations, $C_{T}>1$. Therefore, the frequent update of the Q-network *Q* will have less effect on the target Q value $\hat{Q}$, and the training process will be more stable.

To break down the correlation between training samples, the experience replay technique is proposed by [12]. For each time step t, an experience sample is gathered and stored by the agent into a data set $E=\left\{e_{1},\ldots e_{t}\right\}$, where $e_{t}=\left(s_{t},a_{t},r_{t+1},s_{t+1}\right)$. For each learning iteration, several samples are taken randomly as a mini-batch $\left(s_{j},a_{j},r_{j+1},s_{j+1}\right)$ to perform mini-batch gradient descent [21], and the loss function can be rewritten as

(15) $$L_{j}\left(\theta \right)={\left(y_{j}-Q\left(s_{j},a_{j};\theta \right)\right)}^{2}={\left(\underset{\mathrm{target}\;Q\;\mathrm{value}}{\underset{︸}{\left(r_{j+1}+\gamma \underset{a\_}{\max}\hat{Q}\left(s_{j+1},a\_;\theta^{-}\right)\right)}}-\underset{\mathrm{estimate}\;Q\;\mathrm{value}}{\underset{︸}{Q\left(s_{j},a_{j};\theta \right)}}\right)}^{2}$$

Finally, a Q learning algorithm using a Q-network as the function approximation is shown in Algorithm 2. The parameter of *Q* and $\hat{Q}$, θ and $\theta^{-}$, are initialized. The target *Q* parameter update period *C_T_*, the experience data set *E*, the ε-greedy policy, the learning rate α, the discount factor γ and the experience replay threshold *p*th are also initialized. The agent then observed the current state $s_{t}$. With a probability ε, the agent will do the action randomly, otherwise, it will choose the action with the largest *Q* value according to the output of the Q-network, i.e., $a_{t}=\underset{a\_}{\arg\max}Q\left(s_{t},a\_;\theta \right)$. After applying an action, the agent observed the reward and the representation of the next state, and $\left(s_{t},a_{t},r_{t+1},s_{t+1}\right)$ will be stored into *E*. After cumulating enough experiences, an experience replay can be performed by sampling a mini-batch of experiences $\left(s_{j},a_{j},r_{j+1},s_{j+1}\right)$ from E randomly. The mini-batch target Q values $y_{j}$ are calculated by the $\hat{Q}$ instead of Q because of the performing of the fixed target Q-network technique, and then a mini-batch gradient descent [21] is applied to minimize the loss function shown in (37). The target Q-network parameter $\theta^{-}$ will be updated for every *C_T_* iterations. Finally, the state is updated, and a new iteration will begin.

**Algorithm 2**: Q-learning using Q-network approximation with fixed target Q-networks and experience replayInitialize *C_T_*, *E*,$\theta ,\theta^{-},\varepsilon ,\alpha ,\gamma ,pth,0\leq \varepsilon \leq 1,0\leq \alpha <1,0<\gamma <1$
Observe $s_{t}$
**Repeat** (for each time step *t*)
$\;\;\left\{\begin{array}{cc} \mathrm{randomly}\;\mathrm{choose}\;a_{t} & \;\;\mathrm{probability}\;\varepsilon \\ a_{t}=\underset{a\_\in A\left(s_{t}\right)}{\arg\max}Q\left(s_{t},a\_;\theta \right) & \;\;\;\mathrm{otherwise} \end{array}\right.$
   Apply $a_{t}$, observe $r_{t+1}$ and $s_{t+1}$
   Store $\left(s_{t},a_{t},r_{t+1},s_{t+1}\right)$ in *E*
   If t>pth
    Randomly Sample mini-batch $\left(s_{j},a_{j},r_{j+1},s_{j+1}\right)$ from *E*   Calculate loss function as (15)    Perform mini-batch gradient descent to optimize the loss function   Every *C_T_* step update $\hat{Q}\leftarrow Q$
  
$s_{t+1}\leftarrow s_{t}$


### 3.3. Design of an RL MPPT System

**Figure 16:**
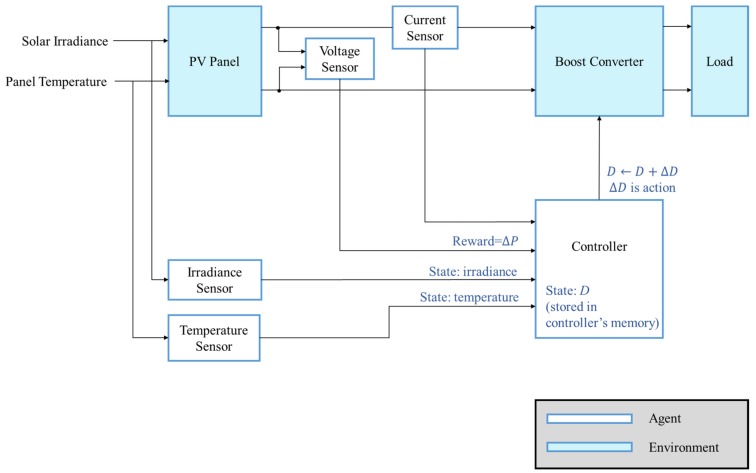
Agent and environment defined in the RL MPPT system.

To perform MPPT based on RL, the system must be able to be described by MDP. The element needed in the RL MPPT system is defined in Table 2. The PV module and the converter can be seen as the environment, and the controller is the agent as depicted in Figure 16. The goal of the agent is to reach the MPP through interacting with the environment.

The system’s condition, the state, is described by the solar irradiance, the module temperature and the duty ratio D since the *I-V* curve is affected by the solar irradiance and the module temperature as mentioned previously. The system’s operating point is at the intersection of the *I–V* curve and the load line, and the load line is controlled by the duty ratio of the converter.

In this study, the action is defined as a set of duty ratio changing step  ∆D with different step sizes. Therefore, the tracking progress can be seen as a sequential decision-making problem, i.e., the MPP of the system can be reached by applying a series of variable step sizes ∆D appropriately.

The reward is a numerical signal that helps the agent judge how “good” or “bad” an action is. The action that moves the operating point close to the MPP is better than the action that moves the operating point away from the MPP. Therefore, the power difference $∆P=P^{\prime }-P$ is defined as the reward since it provides not only the moving direction of the operating point but also the numerical scaling representation of the effect caused by applying the action, for example, a larger step size may lead to a larger power difference.

**Figure 17:**
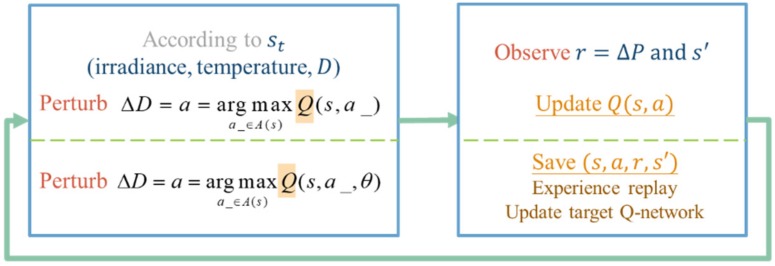
Simple workflow of the RL MPPT.

Also, the Markovian property holds since the current state is only affected by the state and the action taken one step before. Through combining the elements of the MDP model designed in Table 2 and the concept of the RL MPPT shown in Figure 7, the detail of the RL MPPT can be described as Figure 17.

**Figure 18:**
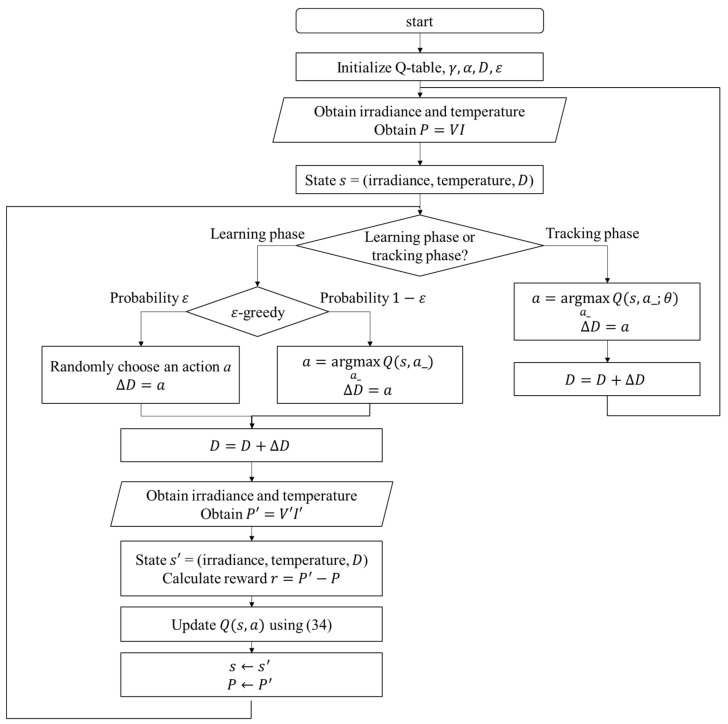
Flowchart of the RL MPPT using the Q-table (RL-QT MPPT).

**Figure 19:**
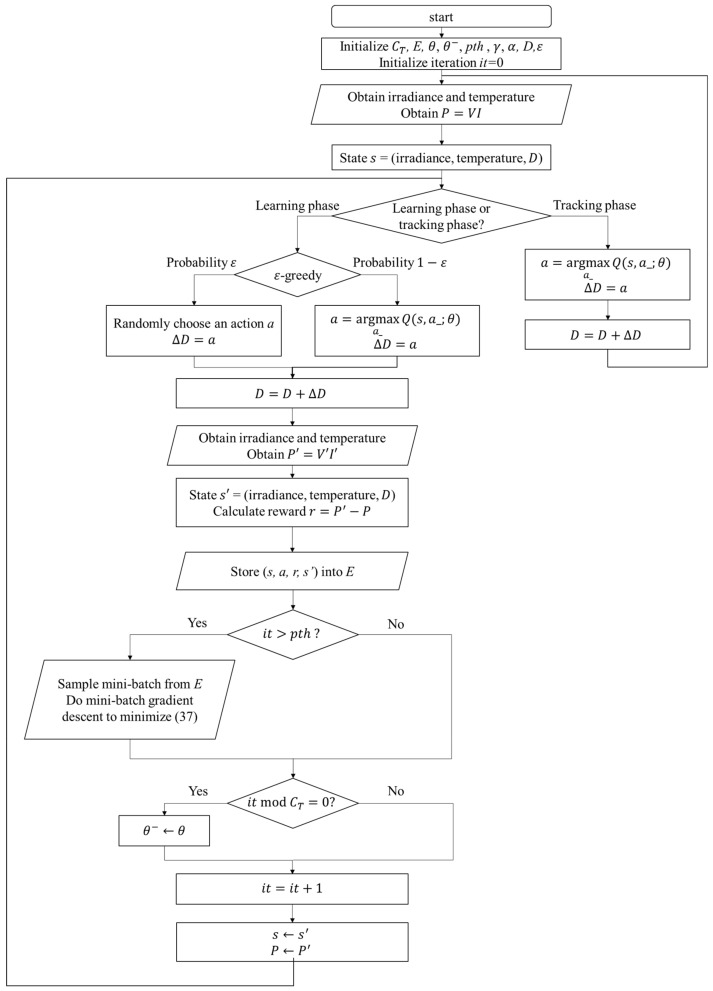
Flowchart of the RL MPPT using the Q-network (RL-QN MPPT).

The perturbation step size is chosen according to the Q value of the current irradiance, temperature, and duty ratio *D*. After applying the change of *D*, the power difference ΔP and the new state description $s^{\prime }$ can be observed. In the Q-table approach, the experience will be used to update the Q value immediately, but in the Q-network approach, it will be stored to perform experience replay. With the simple workflow of the RL MPPT described as above, the flowcharts of the RL MPPT using Q-table (RL-QT MPPT) and the RL MPPT using Q-network (RL-QN MPPT) are shown in Figure 18 and Figure 19. The flowcharts essentially follow the Q-learning algorithms provided in Algorithm 1 and Algorithm 2, respectively.

The proposed RL MPPT methods in this paper include two phases, the learning phase and tracking phase. In the learning phase, the experiences are learned, i.e., the Q-table or the Q-network is updated. However, to speed up the tracking speed, the update process is skipped in the tracking phase. The description of the learning phase and the tracking phase of the RL-QT MPPT and RL-QN MPPT are shown below:

(a) Learning Phase of RL-QT MPPT

First, the Q-table, the discount factor γ, the learning rate α, the ε-greedy policy, and the duty ratio *D* are initialized, and the solar irradiance and the temperature of the PV module are sensed to form the state representation (irradiance, temperature, *D*). The output power of the PV module is sensed and stored as *P*. With a probability of ε, the agent will randomly choose an action and change the perturbation step size. Otherwise it will follow the Q-table and choose the action with the largest Q value. After applying the new duty ratio, the irradiance, the temperature and the output power P′ are sensed again. Therefore, the succeeding state s′ is obtained, and the reward *r* can be calculated as $P^{\prime }-P$. Finally, with $\left(s,a,r,s^{\prime }\right)$, the corresponding element in the Q-table, i.e., $Q\left(s,a\right)$, can be updated by (34).

(b) Tracking Phase of RL-QT MPPT

In the tracking phase, the agent selects the action by looking up the Q-table, and *D* is changed accordingly. Then the iteration ends without updating the Q-table.

(c) Learning Phase of RL-QN MPPT

The RL-QN MPPT is similar to the RL-QT MPPT. First, the fixed target Q-network update iteration $C_{T}$, the experience dataset *E*, the network parameters θ and $\theta^{-}$, the experience replay threshold pth, the discount factor γ, the learning rate α, the ε-greedy policy, and the duty ratio *D* are initialized, and the iteration counter is also initialized. Then the state (irradiance, temperature, *D*) is obtained by acquiring the solar irradiance data and the module temperature data. The output power of the PV module is calculated as P=VI. Under the ε-greedy policy, the action will be chosen randomly in a probability ε, otherwise the action with the largest Q value will be taken. The subsequent state representation $s^{\prime }\;$ and the reward *r* can be obtained after changing the duty ratio of the converter. The experience $\left(s,a,r,s^{\prime }\right)$ is stored in *E*, and if the experiences in *E* are enough, the experience replay techniques will be performed. For every *C_T_* step, the fixed target Q-network weight $\theta^{-}$ will be updated, and finally, it will be increased to move onto the next iteration.

(d) Tracking Phase of RL-QN MPPT

In the tracking phase, the agent follows the policy approximated by the Q-network, i.e., always choose the action with the largest Q value. Then *D* is modified by ∆D. The system’s operating point is changed, but the experience is not stored to speed up tracking. Then a new iteration will begin.

## 4. Results

### 4.1. System Configuration

**Figure 20:**
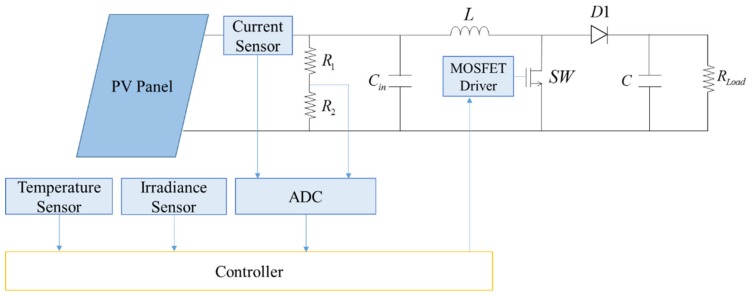
Hardware structure of the proposed system.

The environment configuration of both simulation and experiment is shown in Table 3, including the description of the PV module and the parameter of the DC–DC boost converter. The agent configuration is shown in Table 4, which is identical for both simulation and experiment. The corresponding hardware structure is depicted in Figure 20. For the environment described in Table 3, the configuration of the PV module, the DC–DC boost converter and the resistive load are identical for both simulation and experiment, while two large resistor $R_{1}$ and $R_{2}$ are added into the actual circuit as a voltage divider to measure the voltage, as shown in Figure 20.

As for the configuration of the agent, the range of the duty ratio is set between 0.2 and 0.9 due to the limit of the hardware. The P&O method and the RL MPPT methods are performed one time per second for both simulation and experiment. ΔD of the P&O method is fixed at 0.05, while the RL MPPT methods provide a set of actions with different step sizes, i.e., $A=\left\{\Delta D=0,\Delta D=\pm 0.01,\Delta D=\pm 0.05,\Delta D=\pm 0.1\right\}$. The state, reward, ε-greedy, and the discount factor γ are the same in the RL-QT MPPT and RL-QN MPPT. However, the discretization of the state is needed to perform in the RL-QT MPPT method. For the RL-QT MPPT method, the irradiance is discretized into 10 levels between 0 and 1000 *W*/m^2^, and the temperature is divided into 6 levels, which are below 20 °C, 20~30 °C, 30~40 °C, 40~50 °C, 50~60 °C, and beyond 60 °C, respectively. The level of *D* is discretized by 0.01, the minimum value of non-zero ΔD, and is limited between 0.2~0.9 as mentioned before. Therefore, the size of the Q-table can be obtained by calculating the size of the state space and the action space, which is 4260*7.

A multi-layer perceptron is used as the Q-network in this study. After several attempts and adjustments, a 5-layer structure with 3 hidden layers is applied in both simulation and experiment. The input layer consists of 3 neurons, which are the irradiance, the temperature, and duty ratio input, respectively. Each hidden layer is constructed by 40 neurons, and the output layer has 7 neurons, which represent the Q value of 7 actions,ΔD=0,ΔD=±0.01,ΔD=±0.05,ΔD=±0.1, respectively. The learning rate α is 0.9 in RL-QT MPPT method and 0.0001 in RL-QN MPPT method. $C_{T}$, *E* and *p*th are also initialized for the Q-network method to perform the experience replay and the fixed target Q-network techniques. The total amount of experiences gathered by RL-QN MPPT in the learning phase are 18,646 and 26,838 for the simulation and the experiment, respectively.

### 4.2. Simulation Result

**Figure 21:**
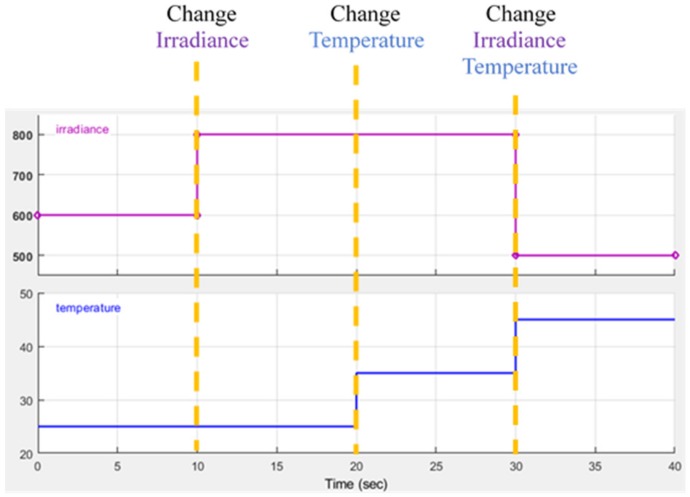
Irradiance and temperature simulation conditions.

**Figure 22:**
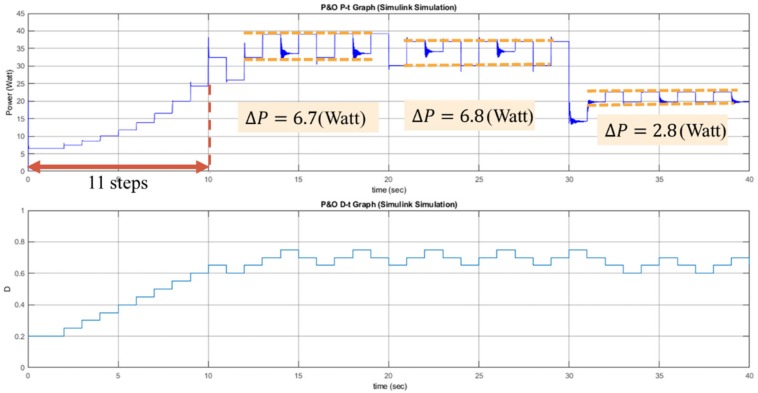
*P-t* graph and *D-t* graph of the P&O simulation result.

**Figure 23:**
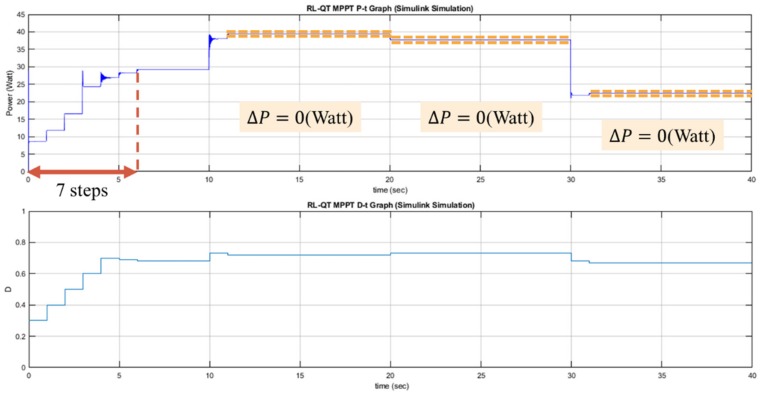
*P-t* graph and *D-t* graph of the RL-QT MPPT simulation result.

**Figure 24:**
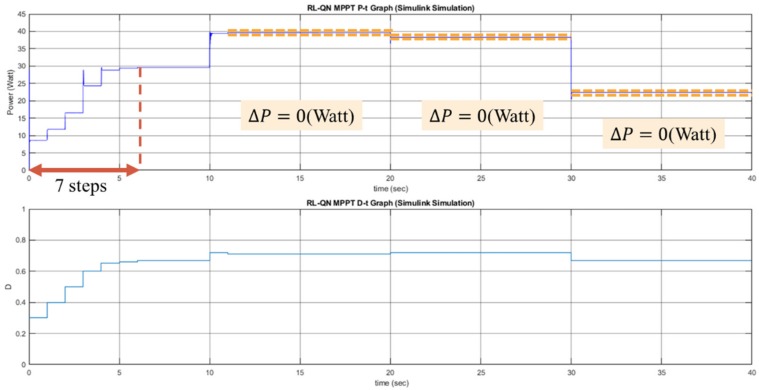
*P-t* graph and *D-t* graph of the RL-QN MPPT simulation result.

The performance of the P&O, RL-QT MPPT, and RL-QN MPPT methods are simulated by MATLAB and Simulink R2017b with AMD Ryzen Threadripper 1920X processor, 3.50 GHz, 64 GB of DRAM memory and Microsoft Windows 10 operating system. With the irradiance and the temperature signal and the simulation configuration provided in Figure 21, Table 3 and Table 4, the *P-t* graph and the *D-t* graph of the P&O method, RL-QT MPPT and RL-QN MPPT methods are shown in Figure 22, Figure 23 and Figure 24, respectively. For the RL MPPT methods, larger step sizes are chosen when the operating point is far away from the MPP, and the smaller step sizes are selected when the operating point is near the MPP. Consequently, the RL MPPT methods outperform the P&O method since both of the RL-QT MPPT and the RL-QN MPPT provide smaller ripples and faster tracking speeds.

### 4.3. Experimental Result

**Figure 25:**
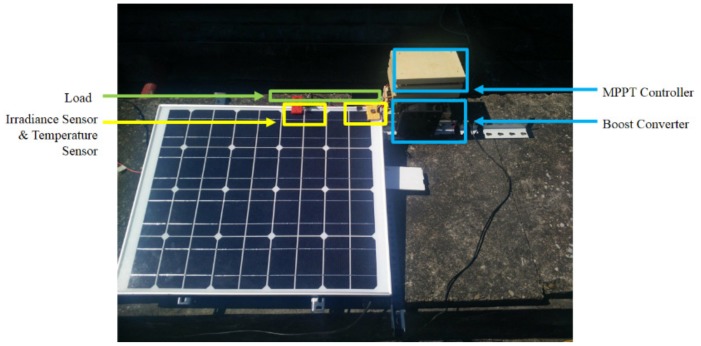
Overview of the hardware setup.

The hardware specification for the actual experiment setup are listed in Table 5. An analog to digital converter (ADC) is needed since there is no built-in ADC in Raspberry Pi 3 Model B [22]. The voltage is measured by the ADC, and the analog output of the current sensor is transferred into the digital signal by the ADC as well. An ambient light sensor module MAX44009 GY-49 is used to measure the illuminance, which can be converted to the solar irradiance with a ratio of 0.0079 *W/m^2^* per lux [23]. To measure the temperature, an infrared thermometer non-contact module MLX90614 GY-906 is used to measure the surface temperature of the PV module. The power MOSFET IRF840 is driven by the photocoupler TLP250. With a high current capability, 1N5408 is an appropriate choice for the diode D1 in the boost converter. Finally, the actual experiment setup is shown in Figure 25.

**Figure 26:**
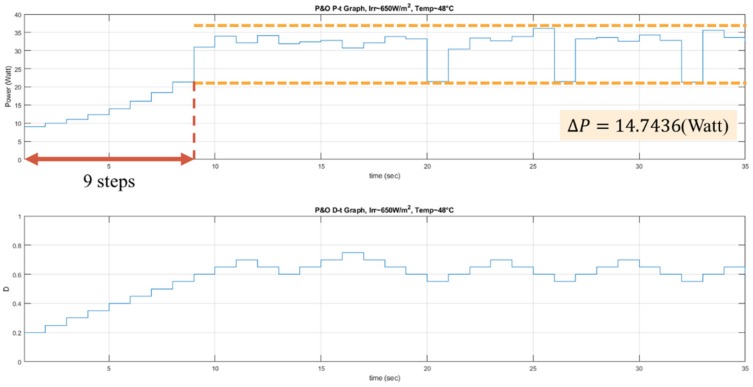
*P-t* graph and *D-t* graph of the P&O experiment result.

**Figure 27:**
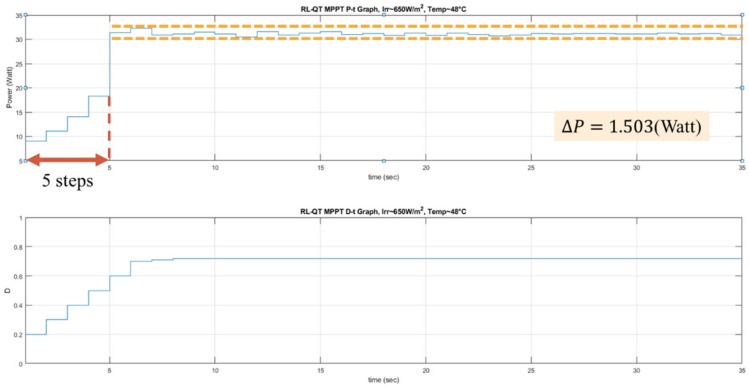
*P-t* graph and *D-t* graph of the RL-QT MPPT experiment result.

**Figure 28:**
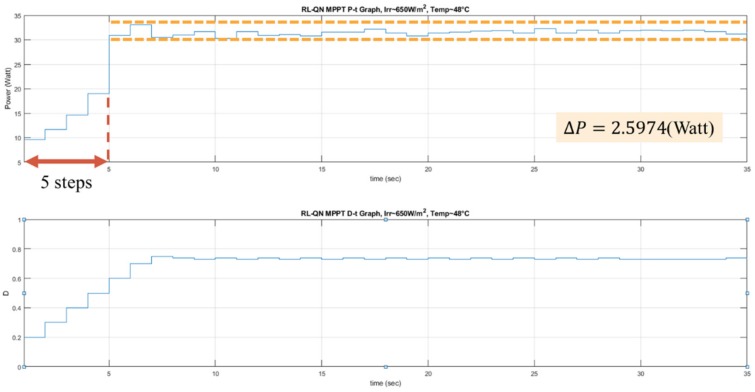
*P-t* graph and *D-t* graph of the RL-QN MPPT experiment result.

The experiments of the P&O method, the RL-QT MPPT method, and the RL-QN MPPT method were conducted under similar environmental conditions. The irradiance was about 650 *W/m^2^*, and the surface temperature of the PV module was about 48 °C. The result is shown in Figure 26, Figure 27 and Figure 28. Similar to the simulation result, the step sizes are selected appropriately by the RL MPPT methods during the tracking process, which leads to a better performance comparing to the P&O method.

Table 6 shows the comparison of the experiment results of the three solar MPPT tracking methods. Both the RL-QT MPPT and the RL-QN MPPT are capable of tracking the MPP with fewer tracking steps than the P&O method and remain stable near the MPP. The average power around MPP of the two RL MPPT methods are near that of the P&O method, which indicate that the proposed methods are able to track the MPP.

## 5. Conclusions

In this paper, two MPPT methods based on model-free reinforcement learning are proposed. The tracking process can be seen as a sequential decision-making problem since the MPP can be achieved through selecting an appropriate perturbation step size for every time step. Therefore, an MDP model is suitable for describing the interaction between the circuit connected to the PV module and the controller which is able to choose ΔD and change the duty ratio *D* of the circuit. An MDP model consists of four elements, which are state, action, transition, and reward. With the MDP model described, an RL-QT MPPT method is proposed by constructing the Q-table to perform MPPT control. However, the state representation is needed to be discretized for the tabular method, which may cause the loss of MPPT control accuracy. Therefore, a Q-network-based MPPT method is proposed. In the RL-QN MPPT method, the Q-table is approximated by a neural network, so that the discretization of the states are not needed. For both RL-QT MPPT and RL-QN MPPT, the tracking method consists of the learning phase and the tracking phase, which is able to expedite the tracking process since the Q value will not be updated in the tracking phase. A conclusion can be drawn from the simulation and experimental results that the RL MPPT methods are more effective than the traditional P&O method to track the MPP since the proposed RL MPPT methods possess smaller ripples and faster tracking speeds. Furthermore, for the proposed RL MPPT methods, the RL-QT MPPT method performs with smaller oscillations and the RL-QN MPPT method achieves a higher average power.

## Figures and Tables

**Table 1:** Q-table example.

*Q*(*s*,*a*)	*A*1	*A*2	*A*3
*S*1	*Q*(*S*1,*A*1) = 0.5	*Q*(*S*1,*A*2) = 1	*Q*(*S*1,*A*3) = 1.5
*S*2	*Q*(*S*2,*A*1) = 3	*Q*(*S*2,*A*2) = −1	*Q*(*S*2,*A*3) = 2
*S*3	*Q*(*S*3,*A*1) = 3	*Q*(*S*3,*A*2) = 5	*Q*(*S*3,*A*3) = 0.5
*S*4	*Q*(*S*4,*A*1) = −1	*Q*(*S*3,*A*2) = −5	*Q*(*S*4,*A*3) = −2

**Table 2:** RL MPPT system element selection.

Parameter Needed to Perform RL	Parameter Selection in Solar MPPT System
Environment	PV module and converter
Agent	controller
State	(irradiance, temperature, duty ratio)
Action	ΔD
Reward	$\Delta P=P^{\prime }-P$

**Table 3:** Environment configuration of simulation and experiment.

PV Module	Power = 50 *W*, Voc = 21.24*V*, Isc = 3.05*A*, (Test Condition: 25 °C, 1000 *W/m^2^*)
***L***	1.1 *mH*
***C***	330 μF
$R_{Load}$	100 Ω
$C_{in}$	1000 μF

**Table 4:** Agent–configuration of simulation and experiment.

	RL-QT MPPT	RL-QN MPPT
***D* range**	0.2~0.9	
**Sampling time**	1 s	
***ΔD* (action)**	{0, ±0.01, ±0.05, ±0.1}	
**State**	(irradiance, temperature, *D*)	
**Reward**	ΔP	
***ε***	1	
***γ***	0.3	
**Q value storing type**	Q-table 4260*7	Q-network 3-40-40-40-7
***α***	0.9	0.0001
***C_T_***	N/A	100
***E***		No limit
***pth***		16

**Table 5:** Hardware specification of the experiment.

Hardware Specification	
**Development board**	Raspberry Pi 3 Model B
**ADC**	ADS1115
**Voltage sensor**	ADS1115
**Current sensor**	ACS723
**Irradiance sensor**	MAX44009 GY-49
**Temperature sensor**	MLX90614 GY-906
**MOSFET driver**	TLP250
**Diode D1**	1N5408

**Table 6:** Comparison of the experimental results.

	P&O	RL-QT MPPT	RL-QN MPPT
**Tracking steps (Sec)**	9	5	5
**Oscillation range (Watt)**	14.7436	1.503	2.5974
**Average power around MPP (Watt)**	31.7478	31.144	31.4977
